# Ocular Involvement in Hereditary Amyloidosis

**DOI:** 10.3390/genes12070955

**Published:** 2021-06-22

**Authors:** Angelo Maria Minnella, Roberta Rissotto, Elena Antoniazzi, Marco Di Girolamo, Marco Luigetti, Martina Maceroni, Daniela Bacherini, Benedetto Falsini, Stanislao Rizzo, Laura Obici

**Affiliations:** 1 Dipartimento Universitario Testa-Collo Rgani di Senso, Università Cattolica del Sacro Cuore, 00168 Rome, Italy; angelomaria.minnella@unicatt.it (A.M.M.); maceronimartina@gmail.com (M.M.); bfalsini@gmail.com (B.F.); stanislao.rizzo@gmail.com (S.R.); 2 UOC Oculistica, Fondazione Policlinico Universitario A. Gemelli-IRCCS, 00168 Rome, Italy; 3 Eye Clinic, San Paolo Hospital, University of Milan, 20142 Milan, Italy; 4 Institute of Ophthalmolgy, IRCCS Fondazione Policlinico San Matteo, 27100 Pavia, Italy; e.antoniazzi@smatteo.pv.it; 5 Former Director “Presidio Ambulatoriale per le Amiloidosi Sistemiche” Fatebenefratelli “San Giovanni Calibita” Hospital, 00135 Rome, Italy; marco.digi@tin.it; 6 Fondazione Policlinico A. Gemelli IRCCS. UOC Neurologia, 00168 Rome, Italy; mluigetti@gmail.com; 7 Dipartimento di Neuroscienze, Università Cattolica del Sacro Cuore, 00168 Rome, Italy; 8 Department of Neuroscience, Psychology, Drug Research and Child Health, Eye Clinic, University of Florence, 50139 Florence, Italy; daniela.bacherini@gmail.com; 9 Amyloidosis Research and Treatment Centre, IRCCS Fondazione Policlinico San Matteo, 27100 Pavia, Italy; l.Obici@smatteo.pv.it

**Keywords:** amyloid, amyloidosis, ATTR, transthyretin, ocular amyloidosis, vitrectomy, vitreous opacities, keratoepithelin, gelsolin, corneal lattice dystrophy, personalized medicine

## Abstract

The term amyloidosis describes a group of rare diseases caused by protein conformation abnormalities resulting in extracellular deposition and accumulation of insoluble fibrillar aggregates. So far, 36 amyloid precursor proteins have been identified, and each one is responsible for a specific disease entity. Transthyretin amyloidosis (ATTRv) is one of the most common forms of systemic and ocular amyloidosis, due to the deposition of transthyretin (TTR), which is a transport protein mainly synthesized in the liver but also in the retinal pigment epithelial cells. ATTRv amyloidosis may be misdiagnosed with several other conditions, resulting in a significant diagnostic delay. Gelsolin and keratoepithelin are other proteins that, when mutated, are responsible for a systemic amyloid disease with significant ocular manifestations that not infrequently appear before systemic involvement. The main signs of ocular amyloid deposition are in the cornea, irido-corneal angle and vitreous, causing complications related to vasculopathy and neuropathy at the local level. This review aims at describing the main biochemical, histopathological and clinical features of systemic amyloidosis associated with eye involvement, with particular emphasis on the inherited forms. We discuss currently available treatments, focusing on ocular involvement and specific ophthalmologic management and highlighting the importance of a prompt treatment for the potential sight-threatening complications derived from amyloid deposition in ocular tissues.

## 1. Introduction

The term “amyloidosis” encompasses different disease entities deriving from conformational changes in native, soluble proteins that misfold and aggregate extracellularly into insoluble, highly ordered fibrils [1]. This occurs especially as a result of a mutation but also secondarily to inflammatory, degenerative and neoplastic processes [1,2,3].

The name *amyloid* comes from the early mistaken identification of the substance as starch (*amylum* in Latin) by Rudolph Virchow in 1854. Virchow, a German physician, examined cerebral corpora amylacea that he discovered to stain pale blue using crude iodine staining and violet after further staining with sulfuric acid [4]. Such an observation led him to assume the analyzed material was cellulose and he named it amyloid. Subsequently, Alan Cohen and Evan Calkins [5] studied the fibrillary structure of different types of amyloid with electron microscopy in 1959. For some time, the scientific community debated whether or not amyloid deposits were fatty deposits or carbohydrate deposits until it was finally resolved that they were neither, but rather a deposition of proteinaceous material.

Amyloid diseases are classified according to the amyloidogenic precursor protein. According to the 2020 amyloid nomenclature proposed by the International Society of Amyloidosis [6], 36 unrelated proteins are known to be associated with amyloid diseases.

The eye is a potential target organ. Proteins that may form amyloid deposits involving the eye include TTR; gelsolin, in which ocular involvement is part of a systemic disease and keratoepithelin and lactoferrin, in which the pathological process is exclusively located in the eye (Table 1). The ophthalmologist plays a crucial role in suspecting, diagnosing and managing these diseases.

In order to properly identify disease-related ocular alterations, a comprehensive ophthalmological visit is of great importance and should include best corrected visual acuity, tonometry, anterior segment slit-lamp examination, optic disc and retina funduscopic examination, corneal confocal microscopy and eventually a visual field test if optic nerve damage is suspected. Almost all ophthalmologic disease manifestations are treatable either with surgery, as in the case of vitreous opacities or anterior capsule opacities of the lens, or with topical therapy, as in the case of glaucoma or keratoconjunctivitis sicca.

This review aims at describing the main biochemical, histopathological and clinical features of systemic amyloidosis associated with eye involvement, with particular emphasis on the inherited forms, focusing on ocular involvement and ophthalmological manifestations. We discuss currently available treatments, including specific ophthalmologic management, highlighting the sight-threatening complications derived from amyloid deposition in ocular tissues.

## 2. Hereditary ATTR Amyloidosis

**Figure 1:**
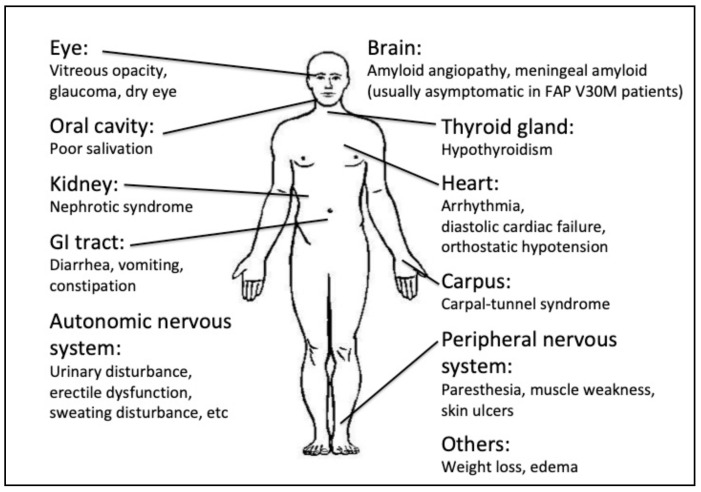
Clinical manifestations in ATTRv patients.

Modified from: Recent advances in transthyretin amyloidosis therapy, Mitsuharu Ueda and Yukio Ando [8].

**Figure 2:**
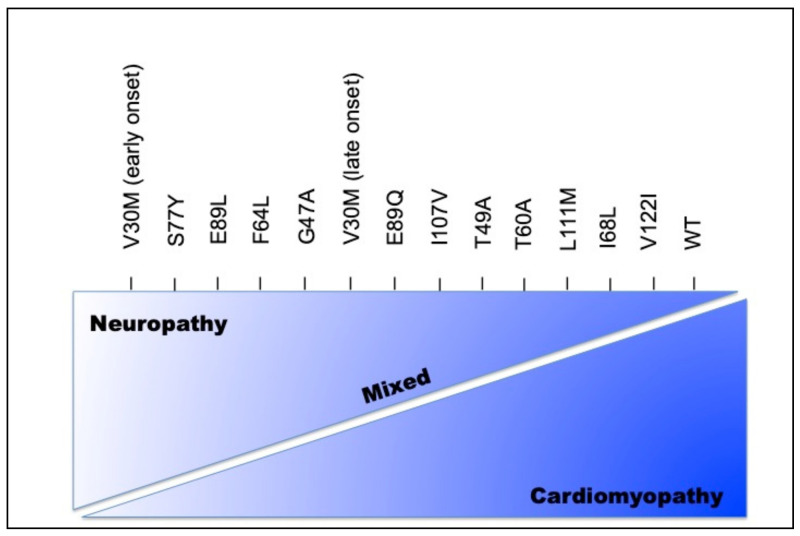
Phenotypic heterogeneity according to genotype.

Modified from: Rapezzi C, Quarta CC et al. Disease profile and differential diagnosis of hereditary transthyretin-related amyloidosis with exclusively cardiac phenotype: an Italian perspective [9].

Hereditary transthyretin amyloidosis (ATTRv), traditionally known as familial amyloid polyneuropathy (FAP), is a progressive autosomal dominant neurodegenerative disease, with variable expressivity, characterized by the accumulation of amyloid in peripheral nerves (somatic and autonomic neuropathy) and the involvement of several organs and tissues, including the eye (Figure 1) [7]. It shows high phenotypic and genotypic heterogeneity, with incomplete penetrance and variable age at onset [8] (Figure 2).

**Figure 3:**
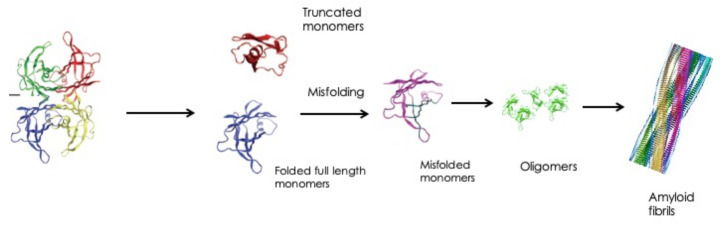
Biochemical mechanisms leading to amyloidosis formation.

Modified from: Recent advances in transthyretin amyloidosis therapy, Mitsuharu Ueda and Yukio Ando [8].

TTR, also known as prealbumin, is a transport protein circulating in plasma at the concentration of 18-45 mg/dL, with variations resulting from age, sex, ethnicity and nutritional status. TTR circulates as a tetramer; however, when exposed to biomechanical forces and proteolytic remodeling, particularly in the presence of mutations that reduce protein stability, it dissociates into full-length and fragmented monomers that are prone to aggregation and subsequent deposition in tissues as amyloid fibrils [10,11] (Figure 3).

More than 130 TTR point mutations have been reported in the literature, with different phenotypes, with Val30Met being the most frequently encountered. This mutation is responsible for the high prevalence of the disease in endemic areas, particularly the north of Portugal, Sweden and Japan [12]. In the Swedish population, the frequency of heterozygosity is approximately 1.5%, with very low penetrance, while in the US, the prevalence of Val30Met mutation in Caucasian ATTRv amyloidosis patients is roughly 1 in 100,000. The most frequently observed mutation in Afro-American, West African and Hispanic subjects is Val122Ile, with a corresponding prevalence of 3.0–3.9%, 5.0% and 0.44%, respectively; the main clinical expression in patients with this mutation is pseudohypertrophic restrictive cardiomyopathy [10,12].

TTR is largely synthesized by the liver, which accounts for about 90% of TTR production [13,14]. In addition to the liver, TTR is also synthesized by retinal pigment epithelium (RPE) cells and choroid plexuses of the brain [15,16]; thus, it is present in CSF where it functions as the most relevant thyroid hormone binding protein and it is thereby thought to transport T4 across the blood–brain barrier.

With regard to the clinical presentation, ATTRv should be suspected when a progressive symmetric axonal sensorimotor polyneuropathy is noted along with one or more of the following features: positive family history for neuropathy, symptoms suggestive of autonomic dysfunction, cardiac involvement (i.e., hypertrophy, arrhythmias, atrioventricular or ventricular blocks and cardiomyopathy), gastrointestinal manifestations, unexplained weight loss, bilateral carpal tunnel syndrome, renal impairment and vitreous opacities. (Figure 1).

The lag time between the onset of symptoms and ATTRv amyloidosis diagnosis can be as long as 6 years [17] due to the diverse clinical manifestations leading to possible misdiagnosis and to the lack of significant family history in many patients from nonendemic countries.

**Figure 4:**
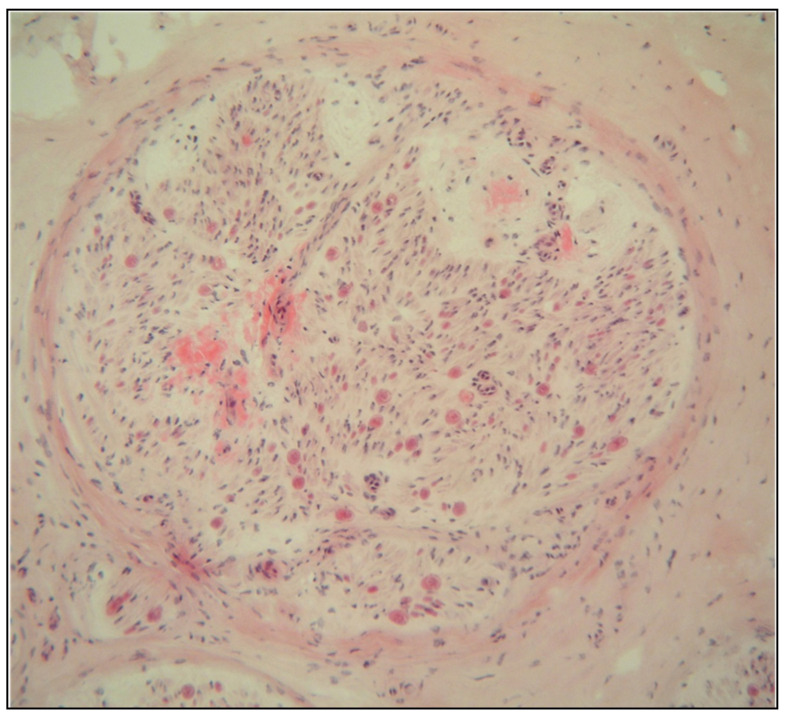
Sural nerve biopsy from a late onset ATTRv patient. H&E showing abundant eosinophilic deposits suggestive of amyloid.

Diagnosis is based on a positive genetic testing and identification of amyloid deposits in a tissue sample, most frequently the salivary gland, abdominal fat, rectum, heart or a peripheral nerve (Figure 4). The diagnostic sensitivity of the biopsy significantly differs depending on the examined tissue and on the various stages of the disease. Nonetheless, pathological characteristics and TTR gene testing, when combined, are pivotal in confirming the diagnosis. Amyloid protein subtype analysis is mandatory when a monoclonal gammopathy of unknown significance (MGUS) coexists. Immunoelectron microscopy and, more recently, proteomics (MS) are the tools of choice for amyloid protein subtype analysis [18].

### 2.1. Eye Involvement in Hereditary TTR Amyloidosis

Among hereditary ATTRv amyloidosis patients, 10% present with ocular involvement which usually appears later in the course of the disease. Some TTR mutations have been clearly associated with eye involvement and are listed in Table 2 (reference in Table).

The eye can synthesize mutant TTR, and progression of ocular involvement after liver transplant is observed even though plasma TTR cannot cross the blood–retina barrier [13]. Thus, ocular manifestations are largely due to the local production of TTR, especially by the retinal pigment epithelium (RPE) [14].

Hara et al. [19] assessed ocular involvement after liver transplantation in ATTR Val30Met patients. A total of 36% developed vitreous opacities, while 18% of patients developed glaucoma. The authors concluded that patients with hereditary ATTR amyloidosis who underwent liver transplantation continued to have a long-term risk of severe ocular manifestations, notably vitreous opacities and glaucoma, with a negative impact on their quality of life [20].

Intraocular TTR synthesis accounts for the progression of vitreous amyloid formation postoperatively, resulting from the local production of mutated TTR in the eye [21,22,23,24]. Differences in biochemical features of amyloid fibrils between vessels in the retina and in extraocular muscles suggest that a different mechanism might underlie amyloid fibrillogenesis in intra- and extraocular tissues [21]. Not only do ocular manifestations in hereditary ATTRv amyloidosis continue to occur after liver transplant, but their development may even speed up after the surgical procedure [19,21,25,26].

Liepnieks et al. [24] provided further evidence that the local synthesis of variant TTR by the RPE is responsible for vitreous amyloid formation. Their study showed that patients with Tyr114Cys and Val30Met mutations developed amyloid vitreous opacities 7 and 14 years, respectively, after liver transplant, thereby lacking the systemic source for variant TTR. They concluded that vitreous amyloid had been synthesized locally since it was largely composed of variant TTR [24].

Nevertheless, Haraoka et al. [14] found that TTR retinal deposits were unexpectedly located in the inner retinal layers rather than closer to RPE in a patient with hereditary ATTRv amyloidosis.

Sandgren et al. [27] analyzed data from 48 liver transplant patients and concluded that, given the increase in prevalence of eye manifestations of the disease over time after LT, an organized ophthalmological follow-up is required, especially for early detection of glaucoma that might be asymptomatic at first but still causes irreversible damage. Due to the prolonged survival granted by transplantation, ocular disease has a higher probability to occur, and there is evidence that liver transplantation may even hasten its appearance.

TTR is widely distributed in the ocular tissues of affected patients. In fact, amyloid deposits have been identified in the corneal endothelium, lens capsule, iris epithelium, retinal pigment epithelium (RPE), ciliary pigment epithelium (CPE), vitreous body, conjunctiva, trabecular meshwork, lacrimal glands and retinal nerve fibers [28].

The first approach to search for TTR amyloid ocular involvement is a complete ophthalmological examination, including a measurement of visual acuity, biomicroscopy with pupil examination, corneal confocal microscopy, anterior chamber and funduscopic examinations, tonometry and, when appropriate, visual field test [28].

Beirão et al. [28] reviewed the medical records of 513 patients affected by ATTR Val30Met amyloidosis, 36 of which were asymptomatic carriers. None of the subjects in the carrier group showed ocular involvement, and the authors suggest performing an ophthalmological evaluation on a biennial basis after a first baseline visit [28].

Due to the nearly ubiquitous deposition of mutated TTR in ocular tissues, ophthalmological manifestations are the most varied and include vitreous opacities, chronic open-angle glaucoma (COAG), abnormal conjunctival vessels (ACVs), sicca keratoconjunctivitis (SKC), loss of corneal sensitivity and neurotrophic corneal ulcers, lens anterior capsule opacities, retinal vascular changes, pupillary light-near dissociation, irregular pupil and optic neuropathy, each one with its related complications, frequently leading to severe vision loss if there is a diagnostic delay. Some of these manifestations can lead to irreversible blindness and have a detrimental impact on patients’ quality of life, despite the possibility of systemic symptoms being stabilized after liver transplantation.

In 1997, Ando et al. [29] analyzed and followed up with 37 Japanese patients with hereditary TTR amyloidosis. The main ocular manifestations were abnormal conjunctival vessels (75.5%), pupillary changes (43.2%), keratoconjunctivitis sicca (40.5%) and glaucoma and vitreous opacities (5.4%). They observed that most ocular manifestations appeared only after liver transplantation [29].

Val30Met mutation often causes an occlusive distal capillaropathy, resulting in different degrees of peripheral retinal ischemia, up to severe pictures with neovascularization. Both Val30Met and non-Val30Met mutations have been previously associated with anterior segment neovascularization and neovascular glaucoma [30].

The severity of ocular manifestations does not necessarily parallel the systemic symptoms, the eye being capable of local TTR production. However, some ocular findings may also be secondary to circulating TTR instead of local secretion by the retinal pigment epithelium (RPE). Vascular conjunctival abnormalities, for instance, are due to plasma TTR, while vitreous amyloid is considered to be formed by TTR secreted by the RPE [31].

#### Ocular Manifestations and Their Treatment

Several ATTR variants are reported to cause ocular manifestations with amyloid deposition in the eye and are listed in Table 2.

**Table 2:** Mutations associated with ocular involvement according to www.amyloidosismutations.com [32].

Mutation	
Cys10Arg [33]	Leu55Gln [34]
Ser23Asn [35]	Leu55Arg [36]
Val30Met [37]	Leu55Pro [38]
Val30Gly [39]	Leu58Arg [40]
Phe33Cys [41]	Phe64Ser [42]
Phe33Ile [43]	Tyr69His [44]
Arg34Gly [45]	Lys70Asn [46]
Lys35Thr [36]	Val71Ala [47]
Ala36Pro [48]	Gly83Arg [49]
Trp41Leu [50]	Ile84Asn [51]
Thr49Ala [52]	Ile84Ser [53]
Gly53Ala [54]	Ala97Ser [55]
Glu54Gly [56]	Tyr114Cys [57]
Glu54Lys [58]	Val122Ala [59]

(a) *Vitreous opacities*

Vitreous amyloidosis is usually bilateral and asymmetrical. Incidence of vitreous opacities in hereditary ATTRv amyloidosis varies from 5.4% to 35%. Vitreous involvement is fairly common and manifests earlier in patients with Arg34Gly, Tyr114Cys (100%), Thr49Ala and Glu54Lys mutations in comparison with Val30Met patients (24%) [25].

**Figure 5:**
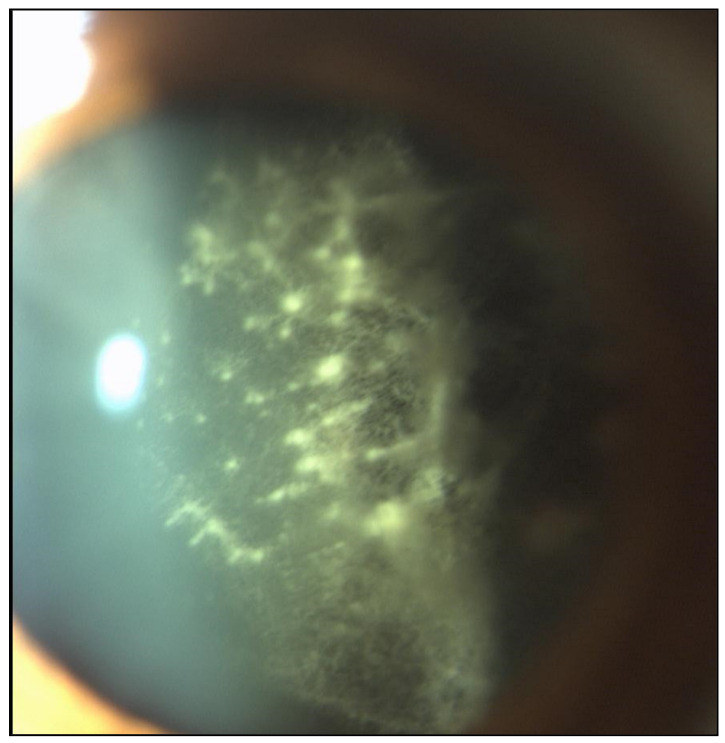
Classical appearance of retrolental vitreous amyloid opacities in ocular TTR amyloidosis examined by slit-lamp examination: multiple sheet-like, cobweb-like and glass wool-like fibrils are present (magnification 16×).

Vitreous collagen fibers act as a scaffold for TTR amyloid aggregation. TTR protein has high affinity for basement membranes, and vitreous matrix is predominantly composed by type 2 collagen, which has structural and biochemical similarities to collagen in basement membranes [3]. The classical appearance of vitreous amyloid has been described as sheet-like, film-like, band-like, cobweb-like, glass wool-like, cotton-like and stringy fibril-like (Figure 5) [3].

Indirect signs of vitreous amyloidosis may be recognized even in asymptomatic patients, and these include pseudopodia lentis (specific white opacities present on the posterior lens capsule), glass wool appearance of vitreous and perivascular amyloid deposits in the retina [3].

Amyloid deposition in the vitreous, with floaters and a consequent progressive decrease in visual acuity, is almost pathognomonic of hereditary amyloidosis by *TTR* gene mutation, occurring either during the natural course of the disease or after hepatic transplantation: the greater the density of vitreous amyloid deposition, the higher the degree of visual loss, up to only light perception. Vitreous amyloidosis may be the initial presenting sign in some cases. In these patients it should prompt additional evaluations to investigate possible subclinical systemic manifestations [1].

**Figure 6:**
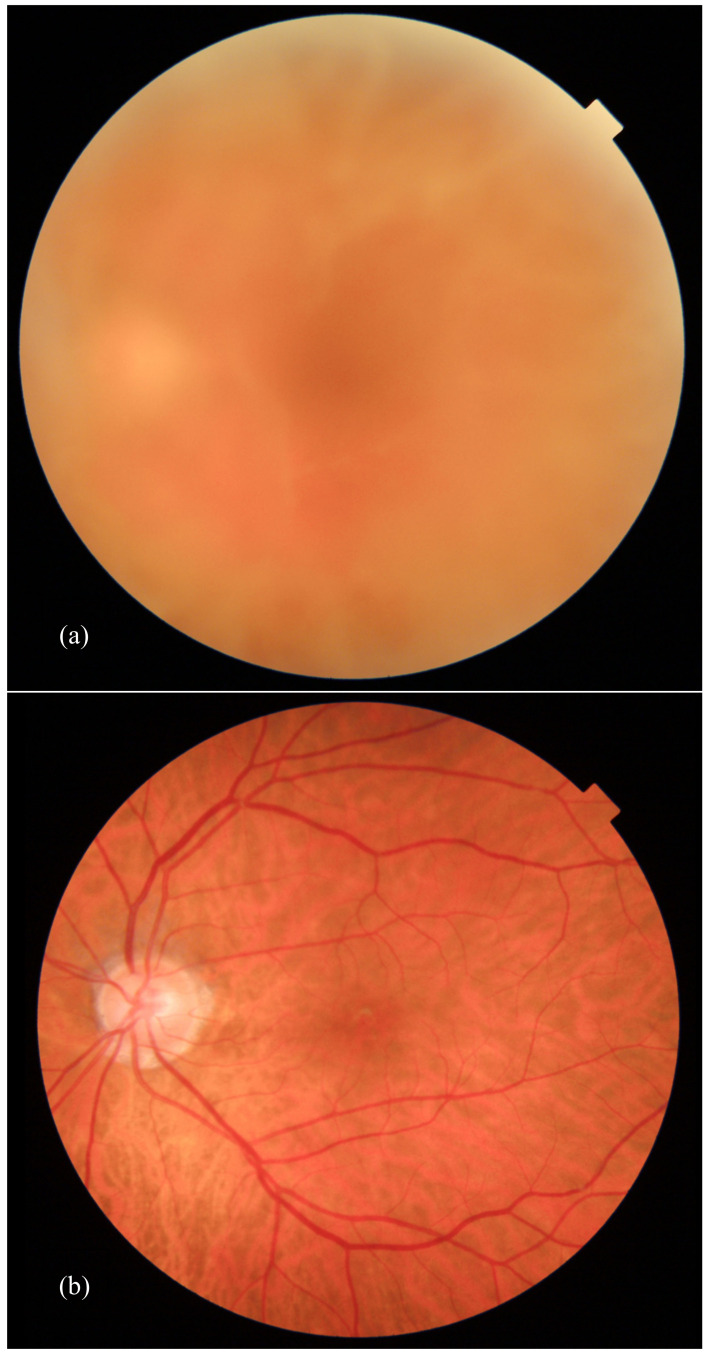
Fundus ophthalmoscopic examination before (**a**) and after (**b**) vitreoretinal surgery (50-degree angle of view).

The current standard treatment is surgical with a 25-gauge pars plana vitrectomy (PPV) [60]. It is crucial to perform a meticulous PPV in order to restore vision, prevent relapses and avoid intraoperative complications (Figure 6). Pars plana vitrectomy is a symptomatic treatment and it is the only treatment available for vision restoration in patients with turbidity due to vitreous opacities. In ATTRv amyloidosis, strong vitreoretinal adhesions are usually present over the retinal vessels, in the area behind the posterior lens capsule and along the vitreous base at the ora serrata. Thus, posterior vitreous detachment can be very hard to perform without causing retinal damage.

Relapsing vitreous opacities due to amyloid redeposition may appear even several years after pars plana vitrectomy, also thanks to the increased life expectancy of these patients, associated with novel therapeutic options. Vitreous amyloid opacities may reoccur due to either dispersion of the residual vitreous deposits or ongoing intraocular production of mutated TTR by the retinal pigment epithelium [61,62].

Vitreous remnants may act as a scaffold for new amyloid material to persist and expand into the vitreous cavity. Relapsing vitreous amyloid may require a second round of surgery with an extensive vitrectomy associated with posterior capsulectomy in order to minimize the chances of recurrence [62].

The relevance of a complete vitrectomy was emphasized by Beirão et al. in a study in which they analyzed the outcomes of extensive PPV, defined as vitrectomy with vitreous removal with indentation, compared to incomplete PPV, without indentation of the periphery. The authors observed no recurrence in the extensive PPV group against a recurrence rate of 69% in eyes submitted to nonextensive vitrectomy, eventually leading to a surgical reintervention in nine eyes of the latter group. They claimed that in all eyes with recurrence of vitreous opacities, new amyloid deposits were present only where vitreous had not been removed [61].

In the same study [61], authors not only highlighted the importance of performing a complete PPV with indentation but also pointed out that proper vitreous removal is facilitated by early PPV, before the appearance of too many pseudopodia lentis (points of adherence of amyloid material on the posterior lens capsule). This also prevents any touching of the posterior capsule with subsequent cataract development, especially in young phakic patients.

Therefore, the best procedure for the accurate removal of opacities is extensive PPV with indentation associated with retrolental vitrectomy, especially in pseudophakic eyes [61].

The removal of amyloid material deposited on the internal limiting membrane (ILM), when present, is necessary in order to avoid recurrence of vitreous opacities. Ferreira et al. [62] performed a second PPV in five eyes of four patients due to relapse of vitreous opacities. They observed that in some of their cases, amyloid deposits on the ILM caused wrinkling of the internal retinal surface. Thus, they performed ILM peeling in the same session in three eyes. Amyloid deposits were described on OCT scans as spindle needle-shaped hyperreflective formations on thickened ILM [62].

Venkatesh et al. [3] performed vitrectomy in ten eyes of five patients affected by ATTRv amyloidosis and vitreous opacities, in order to clear the media haze. They described the vitreous as appearing to be cutting like waxy paper, and they also mentioned that perivascular whitish deposits existed in all the examined eyes [3].

(b) *Chronic Open-Angle Glaucoma (COAG)*

COAG is the leading cause of irreversible blindness in ATTRv subjects.

Pathophysiological mechanisms causing intraocular pressure (IOP) increase include perivascular amyloid deposition in conjunctival and episcleral tissues, intratrabecular deposition and deposition of amyloid on the pupillary edge; these findings may precede glaucoma by months or years [25].

Specifically, glaucoma in ATTRv amyloidosis is mainly due to amyloid protein released into the aqueous humor causing trabecular meshwork obstruction or to raised episcleral venous pressure. Due to these conditions, long-term monitoring and follow-up of IOP in these patients is essential [25].

In glaucomatous patients, erythropoietin (EPO) levels in the aqueous humor are increased compared to baseline, exerting a protective effect on photoreceptors, RPE and ganglion cells. This does not occur in patients with COAG and ATTRv amyloidosis. Thus, since neuroprotective substances are inadequate in these subjects, a more aggressive hypotonic treatment is needed to preserve vision [25].

(c) *Abnormal Conjunctival Vessels (ACVs)*

These harmless changes can be found in almost all patients during the course of the disease and are represented by segmental and fusiform dilatation of conjunctival vessels and sometime subconjunctival hemorrhages [63] and yellow subconjunctival deposits [64].

As opposed to vitreous opacities, ACVs are not due to local TTR production in the eye but derive from circulating mutated protein. Therefore, there is no progression after liver transplant. Conjunctival lymphangectasia [65,66] was recently described.

(d) *Keratoconjunctivitis Sicca (KCS) and Corneal Neuropathy*

**Figure 7:**
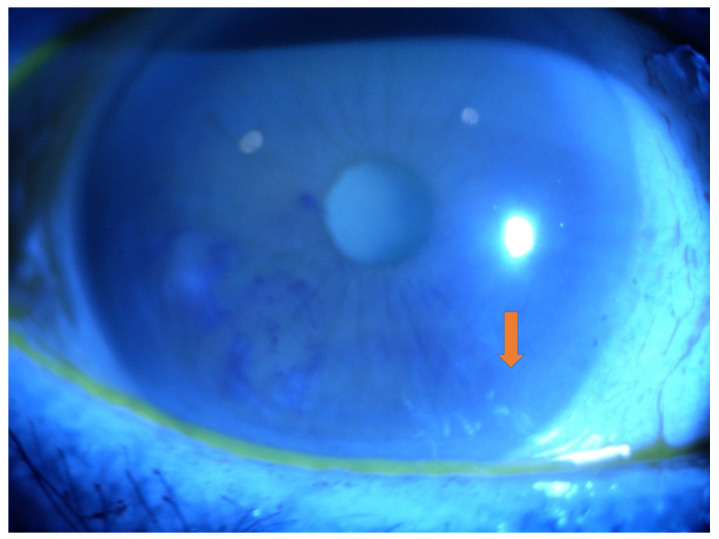
Slit-lamp examination showing areas with altered fluorescein distribution (BUT < 10 sec) and localized fluorescein staining (arrow) in amyloidosis patients (magnification 10× and diffuse illumination).

Keratoconjunctivitis sicca (KCS) is caused by the deposition of amyloid in the corneal layers, damaging epithelium and stroma and altering sensory innervation, with progressively reduced corneal sensitivity (Figure 7).

Moreover, amyloid deposition in the lacrimal gland and autonomic neuropathy contribute to neurotrophic keratopathy with eventual corneal perforation due to eye dryness. Neurotrophic keratopathy is a sight-threatening degenerative corneal disease, characterized by corneal hypoesthesia, spontaneous epithelial breakdown and impairment in corneal healing. The low or absent corneal sensitivity occurs together with reflex tearing, blinking and foreign body sensation [25].

Corneal damage can also be detected at a presymptomatic stage with in vivo laser scanning corneal confocal microscopy (CCM), which is a noninvasive, repeatable examination for evaluating corneal innervation, in particular the sub-basal corneal nerve fiber length (CNFL) [67].

Rousseau et al. [67] applied this concept for studying corneal nerves in patients with ATTRv amyloidosis, concluding that such nerves are damaged in patients with the condition, as demonstrated in other acquired and inherited small fiber neuropathies, namely diabetes [68], Fabry disease [69] and Charcot–Marie–Tooth neuropathy type 1 [70].

**Figure 8:**
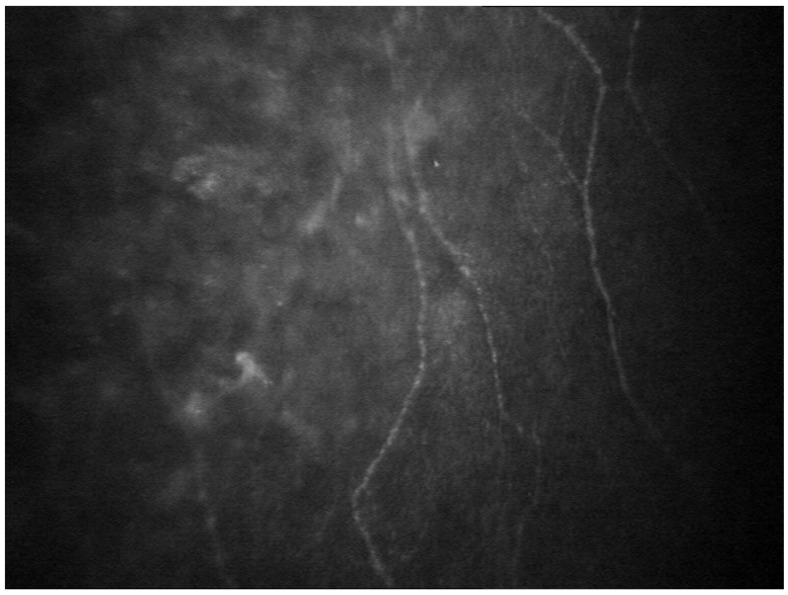
Poorly represented corneal nervous plexa with corneal confocal microscopy in amyloidosis.

Reduced corneal nervous plexa examined by in vivo corneal confocal microscopy are shown in Figure 8. Therefore, CCM could be employed as a useful and sensitive marker of denervation in ATTRv amyloidosis, with diagnostic advantages compared to preexisting methods, and also potentially useful to monitor disease progression in patients receiving new therapies [67].

Treatment for KCS consists of lubricant eye drops in order to avoid friction on the dry cornea. In very severe cases of corneal injury due to TTR deposition, such as in the case of corneal perforation, penetrating keratoplasty (PK) may be a treatment option, even though it does not prevent a relapse because corneal amyloid deposition persists after PK [25].

(e) *Accommodation Defects*

Intraocular amyloid deposition also occurs in the anterior lens capsule. This phenomenon is usually asymmetrical in the two eyes in ATTRv amyloidosis. It determines an impairment in spatial contrast sensitivity and results in early cataract or cataract progression and early presbyopia. The reasons for this are the loss of lens elasticity and the autonomic neuropathy, which jeopardizes the ciliary muscle accommodation.

In a Portuguese study [71] in 144 subjects with hereditary ATTRv amyloidosis, the age of onset of presbyopia was significantly lower than in control individuals and was not influenced by liver transplant.

Treatment for cataracts is surgical and consists of standard phacoemulsification with intraocular lens implantation.

(f) *Chorioretinal Vascular Changes*

As happens for the systemic vasculature, retinal and choroidal vessels are also pathologically involved in amyloid deposition, especially in the endothelial layer [72,73]. Histopathologically, it is possible to observe capillary occlusions and loss of endothelial barrier integrity due to direct damage to endothelial cells.

Hemorrhages and cotton wool spots are the visible signs of such vascular changes, and they occur in about 20% of subjects with hereditary ATTR amyloidosis, with a higher prevalence in cases with the Tyr114Cys mutation whereby retinal venous occlusions may also happen [25].

Chorioretinal vascular changes include retinal amyloid angiopathy (RAA) and choroidal amyloid angiopathy (CAA) [74]. Retinal and choroidal vessel involvement is highlighted by fundus fluorescein angiography and indocyanine green angiography, respectively, rather than by ophthalmoscopic examination.

The most common feature of RAA is retinal microaneurysms, followed by retinal hemorrhages, different degrees of focal retinal ischemia, neovascular glaucoma and preretinal neovascularization. The most severe ocular complication related to RAA in these patients is neovascularization, usually in the anterior segment and, less frequently, in the posterior segment, possibly leading to blindness.

Rousseau et al. [30] used fluorescein angiography and indocyanine green angiography to study retinal and choroidal changes in Val30Met patients. They described different patterns of CAA based on the extent of late hypercyanescence along the choroidal arterial vasculature. The more severe the disease, the more extensive the late hypercyanescence. Moreover, in CAA, the most affected vessels were arteries rather than veins [30].

An association between vitreous amyloidosis and retinal angiopathy also seems to occur. The appearance and rapid progression of amyloidotic retinal angiopathy after vitreous amyloidosis removal (personal unpublished data) suggest that amyloid impregnation of the small terminal vessels in the peripheral retina moves from the vitreous to the vessel lumen and may cause progressive changes to the vessel wall with subsequent obliteration. Thus, its pathophysiology is similar to cerebral amyloid angiopathy, which also continues to progress after liver transplantation. Both seem to be dependent on the vitreous and cerebrospinal fluid mutant TTR and not on circulating mutant TTR.

It is important to closely monitor the peripheral retina with fluorescein angiography and indocyanine green angiography in patients with a long duration of disease in order to timely detect areas of ischemia for prompt therapy. The preferred treatment is laser photocoagulation in peripheral ischemic areas in order to reduce the neoangiogenic drive that may lead to sight-threatening complications, such as neovascular glaucoma, as described by Rousseau et al. [30].

Moreover, retinal laser photocoagulation can be employed in order to suppress ATTR production by destroying the RPE.

(g) *Pupillary Abnormalities*

**Figure 9:**
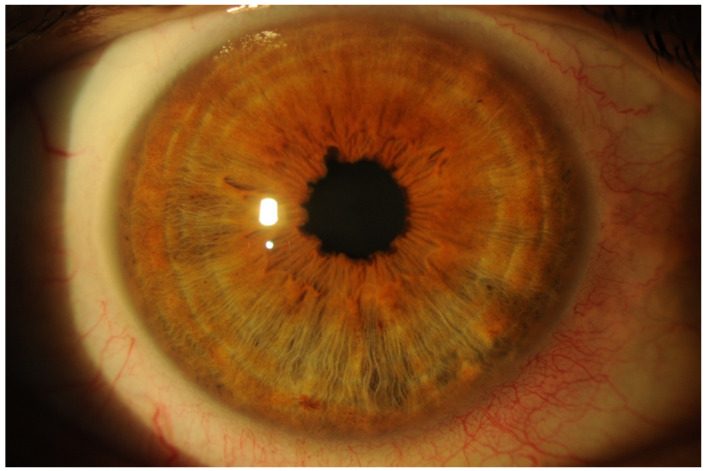
Slit-lamp examination of pupillary indentations considered as a pathognomonic sign of ocular amyloidosis caused by amyloid deposition at the inner pupillary margin (magnification 10× and diffuse illumination).

Peculiar pupillary indentations (Figure 9) are considered a pathognomonic sign of ocular amyloidosis and are due to amyloid deposition at the inner pupillary margin [75].

Pupillary abnormalities, such as anisocoria and pupillary light-near dissociation, are additional frequent findings, but their pathogenesis remains unclear. They might be due to amyloid deposition in iris sphincter muscle or to infiltration of amyloid in the ciliary ganglion [76], thereby causing a reduction in parasympathetic innervation of the iris. In order to preserve sight, it is crucial to detect pupillary changes since amyloid deposition in the pupillary rim may precede the insidious onset of glaucoma by about two years.

(h) *Optic Neuropathy*

A rare but blinding complication of hereditary ATTR amyloidosis is optic neuropathy. Optic nerve involvement is more frequently secondary to uncontrolled glaucoma, but it may be due to ischemic damage caused by amyloid infiltration of blood vessels supplying the nerve [76]. Ischemic optic neuropathy in a patient with ATTRv amyloidosis should be suspected in any case of unexplained progressive visual loss when vitreous opacity and optic nerve damage due to glaucoma have been ruled out.

Therefore, it is important to keep in mind that progressive visual loss due to optic nerve damage is not always due to sustained high intraocular pressure but might be ischemic in nature, meaning it may be related to amyloid vasculopathy and subsequent insufficient blood supply to optic nerves.

(i) *Ocular Adnexal Amyloidosis (OAA)*

Amyloid fibrils can be found in virtually all intra- and extraocular tissues. With regard to tissues surrounding the eye, it is known that amyloid material can be detected in different ocular adnexa, in particular orbits, extraocular muscles, levator palpebrae muscle, eyelids and the lacrimal system [77,78], causing multiple but rare occurrences [79,80,81,82,83]. The variety and infrequency of presentations, accompanied by vague symptoms, result in delayed or missed diagnoses.

OAA can be diagnosed with a biopsy showing amorphous eosinophilic material in the tissues. Histopathological properties are the same as for tissue samples from other body regions, namely apple green birefringence under polarized light after staining with Congo red. Nevertheless, the gold standard is electron microscopy, which, although infrequently employed, allows amyloid fibrils to be correctly identified on the basis of their morphological characteristics.

A Japanese study [14] analyzed nine autopsied eyes from patients with ATTR Val30Met amyloidosis, three of which were from Japanese patients and six from Swedish subjects. The purpose of this investigation was to elucidate the distribution of amyloid deposits in ocular tissues. They found out that the frequency of amyloid distribution was 85.7% in extraocular muscles and 44.4% in the sclera, and orbital adipose tissue from all samples was positive for amyloid (100% of analyzed eyes). Moreover, amyloid fibrils were found in the conjunctiva, iris, trabecular meshwork and vitreous body with a frequency of 88.9%, 44.3%, 11.1% and 11.1%, respectively, in the nine autopsied eyes [14].

### 2.2. Systemic Treatment

**Figure 10:**
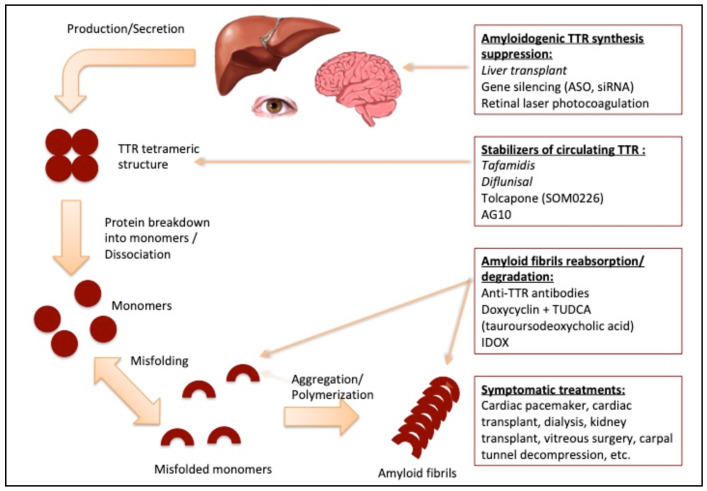
Steps of amyloid formation and corresponding therapeutic strategies. Modified from: Recent advances in transthyretin amyloidosis therapy, Mitsuharu Ueda and Yukio Ando [8].

The therapeutic scenario for hereditary transthyretin amyloidosis has evolved significantly in recent years. Current treatment options are available and they target different steps of the TTR fibrillogenesis process (Figure 10), aiming at halting or slowing disease progression and supporting the dysfunctional organs.

(a) *Liver Transplantation*

The first and only treatment available for ATTRv amyloidosis has long been liver transplantation (LT), aiming at replacing the mutant protein with the wild-type one. This procedure has shown excellent results in increasing 15-year survival in early onset (<50 years old) ATTRv Val30Met patients up to 80%, while its outcome has not been similarly satisfactory in non-Val30Met and late-onset patients. As discussed before, the relevant concern in patients with long survival after LT is cerebral amyloid angiopathy, due to leptomeningeal TTR amyloid infiltration, which leads to possible cognitive decline. Ocular manifestations also complicate the long-term follow-up of LT patients.

(b) *Gene Silencing Agents*

Presently, new and less invasive therapeutic options are available that effectively suppress the concentration of the circulating amyloidogenic precursor TTR, both in its mutant and wild-type form. Two approaches have been investigated in two randomized, placebo controlled, phase 3 clinical trials: the antisense oligonucleotide (ASO) inotersen and the RNA interference agent (RNAi) patisiran. These drugs have been shown to halt disease progression in a substantial proportion of treated patients and even revert neurological manifestations in some cases.

Inotersen is an ASO that has been shown to reduce the circulating TTR concentration by 80% compared to its baseline level. In the NEURO-TTR study, inotersen significantly reduced neurological progression and preserved quality of life compared to placebo after 15 months of treatment [84,85].

Patisiran is the leading drug of the new class of RNAi agents [86], and its intravenous delivery has proven to be effective in suppressing hepatic TTR expression and in cutting down the circulating TTR levels by more than 80% [87]. The APOLLO trial has shown significant stabilization/improvement of neurological disease in patients treated with patisiran compared to placebo.

(c) *TTR Stabilizers*

Another group of drugs acts on the circulating TTR protein by stabilizing its native tetrameric structure, preventing misfolding and further deposition into tissues (TTR stabilizer drugs), to halt or slow disease progression. Oral TTR stabilizers presently include tafamidis, which represent the first drug approved for ATTRv amyloidosis, and diflunisal, both binding to the TTR thyroxine binding sites. Other stabilizers are currently under investigation: tolcapone and AG10.

Tafamidis 20 mg/day is approved for FAP stage I ATTRv polyneuropathy in Europe and in several other countries. Its effect on cardiomyopathy has been confirmed in a phase III trial (ATTR-ACT) published in the NEJM in 2018 [88]. The drug has been recently approved by the FDA and the EMA at a dose of 61 mg for ATTR cardiomyopathy both wild-type and hereditary.

Diflunisal is a nonsteroidal anti-inflammatory drug (NSAID) repurposed for ATTRv amyloidosis at a dose of 250 mg twice daily. Diflunisal was shown to slow neurological progression in both ATTRv Val30Met and non-Val30Met patients [89]. Diflunisal is not formally approved for this indication, but it is available worldwide as an off-label treatment. Its use may raise safety concerns in the long run due to NSAID side effects, and it may be contraindicated in patients with congestive heart failure, concomitant use of anticoagulants and renal insufficiency.

Unfortunately, there is no drug designed for halting intraocular amyloid production. Therefore, ocular manifestations may only benefit from symptomatic treatments, which might also be curative for some specific symptoms, as occurring with vitrectomy for vitreous opacity.

## 3. Hereditary Gelsolin Amyloidosis

Hereditary gelsolin amyloidosis (AGel) is also known as Meretoja syndrome and corneal lattice dystrophy type II [90,91,92]. Jouko Meretoja was the first to report familial amyloidosis of the Finnish type in 1969. He studied ten patients belonging to three different families of Finnish descent who showed a high similarity in clinical symptoms. It is estimated that more than a thousand carriers are present in Finland currently. Additionally, affected subjects have been identified in several countries worldwide since the first report of the disease [90,91,92].

AGel is a monogenic systemic disease inherited in an autosomal dominant fashion. Its pathogenesis results from gain of function mutations in plasma gelsolin that trigger the aggregation of mutated fragments of this protein in affected organs, such as the cornea, peripheral nerves and the skin. Its main features are corneal lattice dystrophy, neurodegeneration with cranial and peripheral neuropathy and cutis laxa [91].

AGel is caused by mutations occurring at low frequency in different populations. There are no reports of a sporadic or wild-type form of gelsolin amyloidosis; hence, mutations are critical. All Finnish AGel patients who have been genetically characterized show the same gelsolin mutation [92]. It is possible that all cases may have a common origin in some populations.

The pathogenic Asp214Asn and Asp214Tyr variants abolish a calcium-binding site in a protein domain, leading to loss of protein stability [93]. As a consequence, the domain unfolds and becomes susceptible to furin cleavage. The product of furin cleavage is a C-terminal 68 kDa fragment that is secreted and subsequently cleaved by proteases (i.e., membrane type 1 matrix metalloprotease) in the extracellular matrix, resulting in 8 and 5 kDa amyloidogenic fragments that are deposited systemically [91].

The 8 and 5 kDa amyloidogenic fragments aggregate by a nucleated polymerization kinetics in which amyloidogenesis is accelerated by oxidized lipids as well as components of the extracellular matrix [91].

Diagnosis is based on gelsolin gene sequencing and on a tissue biopsy. Congo red staining is employed to detect gelsolin amyloid deposition in tissue specimens.

Presently, no specific therapy is available for AGel, but symptomatic treatments often improve the quality of life in a substantial way. Management of AGel patients is mostly focused on alleviating symptoms as opposed to targeting the underlying molecular mechanisms leading to the pathology [91].

### 3.1. Clinical Manifestations

The classical form presents in the third decade of life with slowly progressive ophthalmological, neurological and dermatological manifestations, the most common being corneal lattice dystrophy, cranial neuropathy and cutis laxa (Table 1). Less frequent signs of systemic involvement may occur later in the course of the disease, including cardiac, renal and pharyngo-oro-dental abnormalities [94].

A wide range of disease severity can be observed, ranging from mild sensory impairment to profound sensory ataxia with impaired gait, from slight visual impairment to complete blindness and from borderline laxity of facial skin to a major aesthetic handicap [94].

#### 3.1.1. Ocular Involvement

Frequently, patients complain of eye dryness, photophobia, general irritability and periodical visual impairment particularly in the morning, often progressive with age [94].

Histopathology shows deposition in the cornea (especially in the anterior and middle stroma), conjunctiva, sclera, ciliary body, optic nerve sheath and choriocapillaris. Since the amyloid is found in both vascular and avascular structures, it is believed that local synthesis of gelsolin and cleavage into misfolding short fragments, in particular in the cornea, contribute to ocular pathology in AGel [94].

**Figure 11:**
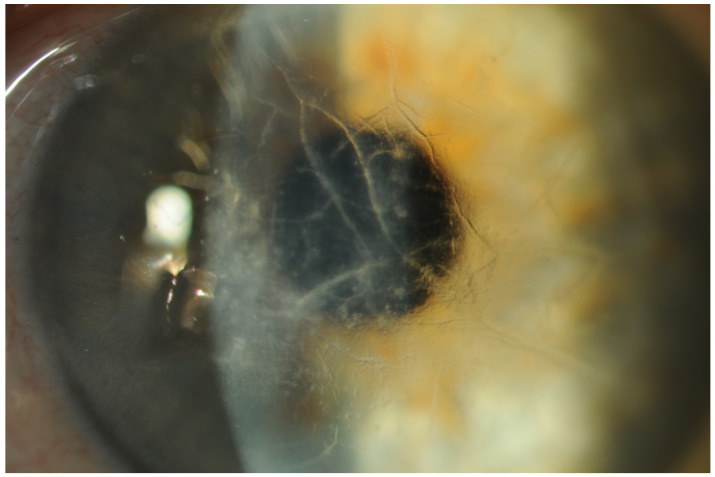
Slit-lamp examination of corneal lattice dystrophy in amyloidotic patients (magnification 16×).

Ophthalmological involvement is generally the first symptom in gelsolin amyloidosis, followed by polyneuropathy, facial nerve palsy and cutis laxa [92]. Corneal lattice dystrophy type II, or gelsolin-related CLD, is a slowly progressive, bilateral disease that causes recurrent corneal erosions, resulting in decreased vision attributable to corneal opacification (Figure 11). It was first described by Meretoja in 1972 as amyloid deposits typically affecting the peripheral cornea in a lace-like fashion, leaving the central cornea roughly intact. The discriminating factor between gelsolin-type CLD and other hereditary corneal dystrophies is the specific pattern of amyloid deposits in the cornea combined with systemic amyloid disease [92,94].

#### 3.1.2. Other Clinical Manifestations

Facial palsy in AGel differs from Bell’s palsy as it is a peripheral palsy of the area innervated by the upper branch of the facial nerve, followed by a slow progression to the area innervated by the lower branches. It may sometimes begin as asymmetrical palsy, but it usually progresses to facial diplegia. Furthermore, other cranial nerves are frequently affected, among which is the trigeminal nerve, causing reduced sensitivity in the head and neck region and contributing to a decrease in corneal sensitivity that, together with corneal amyloid deposition, causes a reduced or absent corneal reflex. Late stages are often characterized by involvement of the glossopharyngeal and hypoglossal nerves, resulting in tongue atrophy and fasciculations, dysarthria and drooling [94].

Dermatological abnormalities result from gelsolin amyloid deposition in the skin basement membrane and generally manifest as cutis laxa, which is thickened and loosened skin with reduced elasticity and resilience; patients show a progressive premature facial aging and have a characteristic facies dominated by a marked drooping and mask-like appearance. Cutis laxa substantially alters the facial appearance, limits facial expressions, restricts visual fields and impairs speech and other oral functioning, causing a significant disability to the patient [94].

Reconstructive plastic surgery may be required in case of facial nerve paralysis with consequent eyelid deformities, including entropion, ectropion, blepharochalasis and lagophthalmos. Brow lifts to alleviate brow ptosis and blepharoplasties to remove thickened skin in the eyelids are often performed, along with forehead lifts and facelifts.

AGel can sometimes present with xerophthalmia and xerostomia, mimicking Sjögren’s syndrome and leading to misdiagnosis. In addition to salivary gland amyloidosis and atrophy, facial nerve dysfunction substantially contributes to uncomfortable and disabling oral dryness [94].

Regarding peripheral neuropathy, a primarily sensory neuropathy can be observed, starting in the lower extremities, and these patients frequently suffer from ischemia due to amyloid deposition in the vasculature. Electrophysiological signs of denervation can be present in young clinically asymptomatic subjects. A further component of polyneuropathy in AGel patients is autonomic dysfunction with the main consequence being orthostatic hypotension. Moreover, gelsolin also deposits in blood vessels, compromising arterial compliance and producing further exacerbation of blood pressure dysregulation.

AGel patients should regularly visit neurologists and cardiologists on an annual basis. AGel can manifest with cardiac conduction defects, and pacemaker treatment may be needed before the age of 60.

Patients who are homozygous for gelsolin mutations are susceptible to developing a severe nephrotic syndrome terminating in end-stage renal failure and having an earlier onset of disease and a faster progression [95].

More recently, two additional gelsolin variants, namely Gly194Arg and Asn211Lys, were identified that are associated only with renal amyloidosis. The reasons for this significant phenotypic variability among the gelsolin mutations reported to date is not defined yet [96,97].

Another possible feature of AGel is generalized amyloid angiopathy, involving spinal and brain vessels, resulting in an increased risk of fatal cerebral bleeds. The amyloidotic vessel wall involvement, together with altered platelet activation, may explain why AGel patients have disproportionate bruising after minor trauma and excessive hemorrhages after surgical procedures in comparison with control subjects.

Eventually, further amyloid deposition in vital organs and microvasculature can become life threatening, and the leading causes of death are generally nephrotic syndrome with uremia, cardiac failure, aspiration pneumonia (linked to bulbar muscle dysfunction) and cerebral hemorrhage (deriving from cerebral angiopathy) [91].

### 3.2. Diagnosis

A clinical suspicion of AGel can be made if biomicroscopy evidence of CLD is accompanied by typical bilateral facial palsies and skin laxity [98]. A useful technique is confocal microscopy that, in addition to corneal amyloid deposition, can reveal the loss of corneal nerves and stromal fibrosis, presumably contributing to ophthalmological symptoms. Immunohistochemistry is a useful technique to localize AGel deposits and differentiate gelsolin-related corneal lattice dystrophy (CLD II) from other corneal lattice dystrophies by using antibodies directed against variant gelsolin [98].

### 3.3. Treatment

The ophthalmologist plays a crucial role not only in diagnosing but also in managing the disease. Primary treatment is aimed at relieving discomfort and preventing complications, such as corneal erosions and glaucoma. Given the reduced corneal sensitivity, lubricating eye drops are frequently prescribed for minimizing the risk of mechanical damage to the cornea, particularly since corneal erosions may go unnoticed because patients do not experience them as painful. Aggressive management of dry eye syndrome is of vital importance in LCD type II patients: in addition to preservative-free lubricating artificial tears, protective contact lenses and topical antibiotics may also be necessary. Prophylaxis and therapy of corneal abrasions and erosions are based on ointments containing vitamin A and panthothenol [98].

Subepithelial corneal deposition of amyloid material is a continuous process, eventually leading to permanent blindness, and cannot be halted. Patients may have other manifestations of corneal disease before developing an overt CLD, such as recurrent corneal epithelium breakdown with painful corneal erosions. Frequent corneal erosions accompanied by subepithelial corneal amyloid deposits results in corneal ulcers and permanent vision loss. The only way to restore corneal clearness is keratoplasty, although new amyloid deposits will gradually accumulate in the graft [92].

Frequent complications of gelsolin amyloidosis are chronic open-angle glaucoma and cataracts. It is advisable to carry out close monitoring of intraocular pressure, which, secondary glaucoma, is the most feared complication.

According to Carrwik and Stenevi [98], strict control of intraocular pressure below 12 mmHg is the only chance for postponing keratoplasty in patients with impaired visual acuity. The reason for this is that glaucoma is almost invariably associated with corneal erosions in these patients, and this has an additional deleterious effect on the damaged cornea. In support of this claim, the authors emphasize that elevated IOP is associated with increased corneal haze [98].

## 4. Keratoepithelin Amyloidosis

Keratoepithelin (KE) is one of the amyloidogenic proteins, and the clinical manifestation of the related amyloidotic disease is a blinding corneal dystrophy. In humans, KE is expressed by the corneal epithelial layer and released towards the corneal stroma; it is synthesized by the healthy epithelium, by the endothelium of Fuch’s dystrophy and by endothelial and stromal cells in healing corneal wounds. However, it also has been detected in the lung and in the bladder smooth muscle. The only pathologically involved organ is the eye, and the distribution of this protein in other human tissues remains unclear [99,100].

KE plays a role in extracellular matrix formation during corneal normal development and healing. It is thought to have an important role in corneal integrity and wound healing processes. A proposed explanation for the still unclear molecular mechanisms underlying the biological effects is that KE would mediate cell adhesion mainly by interacting with integrins [100].

Keratoepithelin has been shown to engage with various extracellular matrix proteins and to facilitate cell attachment, migration, proliferation and differentiation. It binds collagen fibers, fibronectin, decorin, biglycan and integrins, interconnecting extracellular matrix components with resident cells in several tissues. Furthermore, KE is linked to collagen type VI in the corneal stroma, and such a protein complex may have an anchoring function between stroma and Descemet’s membrane.

KE is involved in both primary amyloid deposits of hereditary corneal dystrophies and in secondary amyloidosis of the cornea [100].

With regard to the genetics of primary corneal dystrophies, KE is coded by the gene BIGH3, which is part of the transforming growth factor β-induced (TGFBI) gene on chromosome 5q31. The role of KE gene mutations in human chromosome 5q31 has been unequivocally established by molecular genetics as causative for corneal dystrophies, but nevertheless, the pathogenic mechanisms responsible for abnormal protein aggregation still need to be clarified [100].

The molecular analysis of KE-related corneal dystrophies revealed deposits of abnormal protein in the forms of amyloid fibrils and/or nonamyloid amorphous aggregations.

Population analyses have revealed two hot spots for mutations, Arg-124 and Arg-555, associated with corneal dystrophies. In the Arg-124 mutation, four different mutations give rise to four different phenotypes. The Arg124Cys mutation has been linked with lattice corneal dystrophy type 1, granular corneal dystrophy type 2, granular corneal dystrophy type 3 and a variant of granular corneal dystrophy type 1 [100].

Mutations of KE cause hereditary corneal dystrophies that are characterized by the abnormal deposition of amyloid fibrils and/or granular aggregation in the cornea and have been linked to at least 13 clinically and histopathologically distinct autosomal dominant corneal dystrophies. Amyloid deposits are found in many corneal dystrophies, including lattice dystrophy (LCD) type I, IA, II, IIIA, IIIB, IV, V, VI and VII and granular dystrophy type II, also called Avellino dystrophy [99].

Regarding differential diagnosis, along with the primary amyloid deposits, a variety of ocular diseases can develop secondary amyloid deposits, namely trachoma, lepra, sarcoidosis, interstitial keratitis, phlyctenular keratitis, uveitis, chronic post-traumatic inflammation, glaucoma, keratoconus and retinopathy of prematurity. Recently, point mutations in the transforming growth factor-β-induced gene (TGFBI) encoding for the protein KE have been found to be associated with these corneal diseases [99].

Despite numerous studies, an effective therapeutic approach for these corneal dystrophies is yet to be established.

## 5. Lactoferrin Amyloidosis

Another type of amyloidosis involving the eye is primary ALac amyloidosis, previously known as familial subepithelial corneal amyloidosis or gelatinous drop-like corneal dystrophy (GDLCD), owing to its clinical appearance. This localized form of amyloidosis is inherited in an autosomal-recessive fashion, and it presents a higher incidence in Japan compared to western countries [101,102,103,104].

Lactoferrin, a single polypeptide chain of 692 amino acids with a molecular mass of 78.3 kDa, forms amyloid fibrils that build up underneath the corneal epithelium in the absence of systemic abnormalities [105]. This protein is normally present in human milk, granules of polymorphonucleates and other biological fluids, whereas it is scarce or absent in plasma. Moreover, epithelial acinar cells of the lacrimal glands secrete lactoferrin into tears, which accounts for approximately 25% of the total tear protein [102,105]. The main functions of lactoferrin include the inhibition of RNA degradation and the inhibition of bacterial and myeloid growth [105].

Primary ALac amyloidosis derives from pathological variants of the tumor-associated calcium signal transducer 2 gene (TACSTD2), located on chromosome 1p32.1, formerly known as M1S1. A mutation analysis of TACSTD2 gene is required in order to obtain a reliable diagnosis [101,105].

The mechanism of amyloid formation is still unclear. Nevertheless, the phenotypic results of these mutations are a dysfunctioning epithelial barrier that allows lactoferrin-containing tear fluid to penetrate the subepithelial corneal tissue, culminating in local amyloid formation [101,102].

Disease presentation occurs in the first or second decade of life, and it comes with symptoms that include ocular foreign body sensation together with severely impaired visual acuity, photophobia, hyperemia and epiphora. Slit-lamp examination shows hyperfluorescence involving a wide area of the corneal epithelium and the limbus. Subepithelial lesions are seen with direct illumination in the shape of horizontal bands or multiple mulberry-like nodules. Transmission electron microscopy (TEM) shows a disruption in tight junctions of the superficial epithelial layer, whilst antilactoferrin antibodies highlight deposits in the basal epithelium [101,106].

The appearance of corneal opacities divides ALac amyloidosis into four subtypes: mulberry type, band keratopathy type, kumquat-like (Chinese mandarin-like) type and stromal opacity type. It is yet to be defined if these subtypes are instead different stages of the disease [103].

Treatment is surgical and consists of penetrating keratoplasty, deep lamellar keratoplasty or superficial keratectomy to recover visual acuity, in spite of a high rate of relapses [105].

Localized corneal deposition of lactoferrin may also be secondary to chronic ocular inflammation or to a degenerative ocular disorder, namely keratoconus, trachoma, phlyctenular keratitis, bullous keratopathy, interstitial keratitis, syphilis, trichiasis and spheroidal degeneration [107]. As opposed to primary lactoferrin corneal amyloidosis, the secondary form of the disease is not due to a pathological genetic variant. However, clinical features are quite similar in the two.

Slit-lamp biomicroscopy shows a unilateral milky-white soft mass on the cornea, which is located within the corneal epithelial layer, as revealed by anterior segment optical coherence tomography. In later stages of the disease, Bowman’s layer may become affected [101].

Pathological specimens can be analyzed with Congo red staining and seen under polarized light microscopy or studied with immunochemistry using antilactoferrin antibodies [108].

Lamellar keratoplasty, associated with frequent epilation of the cilia when trichiasis is the long-standing causative process, generally results in the recovery of previous visual acuity, in contrast with primary corneal lactoferrin amyloidosis [101].

## 6. Conclusions

The term amyloidosis describes a group of rare diseases caused by protein conformation abnormalities resulting in extracellular deposition and accumulation of insoluble fibrillar aggregates. So far, 36 amyloid precursor proteins have been identified, and each is responsible for a specific disease entity.

Transthyretin (TTR) is an amyloidogenic protein mainly synthesized in the liver, although other organs contribute to its production (brain choroid plexuses, retinal pigment epithelial cells). ATTRv amyloidosis is one of the most common forms of systemic amyloidosis and can be associated with eye involvement.

Ocular involvement occurs frequently in hereditary ATTRv amyloidosis patients, mostly after the onset of systemic manifestations. Eye problems arise from the deposition of amyloid fibrils into ocular tissues, inducing the opacity of dioptric media and causing complications related to vasculopathy and neuropathy at the local level. Ophthalmologists need to be careful and look for subtle ocular findings which characterize this pathology, such as corneal alterations, irregular pupil with amyloid deposits, retrolental and/or vitreous opacities and secondary OAG.

AGel amyloidosis is another systemic amyloid disease in which ocular involvement is an early and pathognomonic finding. Lattice corneal dystrophy type II should raise the suspicion of hereditary gelsolin amyloidosis, especially if accompanied by peripheral facial nerve palsy and peripheral neuropathy.

Keratoepithelin causes exclusively ocular involvement and manifests as heterogeneous corneal dystrophies.

Primary ALac amyloidosis, characterized by corneal deposition of lactoferrin, causes ocular signs and symptoms related to corneal involvement. A secondary form of the disease has been described, but it is not due to a pathological genetic variant.

As discussed in this systematic review, a complete ophthalmological examination is key to detecting and managing ocular involvement, especially when ophthalmic manifestations may be sight threatening.

Moreover, ophthalmologists may play a key role by prompting additional investigations to search for systemic manifestations that may deserve early treatment interventions. This is particularly relevant in ATTRv amyloidosis where the therapeutic armamentarium has extraordinarily expanded in recent years.

## Figures and Tables

**Table 1:** Amyloid fibril proteins and their precursors involved in eye diseases.

Fibril Protein	Precursor Protein	Systemic and/or Localized	Acquired or Hereditary	Target Organs
AL	Immunoglobulin light chain	S,L	A,H	All organs, usually except CNS
AH	Immunoglobulin heavy chain	S,L	A	All organs except CNS
AA	(Apo) Serum amyloid A	S	A	All organs except CNS
ATTR	Transthyretin, wild type	S	A	Heart mainly in males, Lung, Ligaments, Tenosynovium
	Transthyretin, variants	S	H	PNS, ANS, heart, eye, leptomeninges
AGel	Gelsolin, variants	S	H	PNS, cornea
AKer	Keratoepithelin	L	A,H	Cornea
ALac	Lactoferrin	L	A	Cornea

AL, amyloid light chain; AH, amyloid heavy chain; AA, serum amyloid A protein; ATTR, transthyretin amyloid protein; AGel, gelsolin amyloid protein; Aker, keratoepithelin amyloid protein; ALac, lactoferrin amyloid protein; S, systemic; L, localized; A, acquired; H, hereditary; CNS, central nervous system; PNS, peripheral nervous system; ANS, autonomic nervous system. Partially modified from [6].

## Data Availability

Not applicable.

## Methods of Literature Searching

PubMed NCBI was the database used and “Amyloid, amyloidosis, ATTR, transthyretin, ocular amyloidosis, Val30Met, vitrectomy, vitreous opacities, keratoepithelin, gelsolin, hereditary gelsolin amyloidosis, corneal lattice dystrophy, personalised medicine” were the search words used; years covered were 1980–2021 and additional sources (e.g., articles cited in the reference lists of other articles) were not used. The foreign literature was not excluded, and for non-English articles, English abstracts were used. Articles were selected according to journal relevance.
